# BUILDING SOCIAL CAPITAL: LESSONS FROM A SWISS ACTION-RESEARCH INTERVENTION

**DOI:** 10.1093/geroni/igz038.619

**Published:** 2019-11-08

**Authors:** Annahita Ehsan

**Affiliations:** University of Lausanne, Lausanne, Switzerland

## Abstract

Social capital interventions to promote healthy aging seem promising, but recent evidence has questioned how social capital is constructed. In order to understand how social capital is built among older adults, this study draws from the diagnostic phase of one ‘Neighbourhoods in Solidarity’ (NS) intervention, which uses action research to promote wellbeing for older adults (55+) in Swiss communities. These findings arose from ethnographic fieldwork with 77 hours of observation during group gatherings and informal interviews with participants who identified and debated issues in their community. It became evident that the geographic space and the sense of identity that citizens attached to it (herein referred to as ‘place’) played a role in how the NS intervention developed. The community was divided into two groups with distinct identities: one in the north and one in the south. The sense of place for both groups was simultaneously disrupted when outsiders moved to both areas, exacerbating tensions. The NS brought the two groups together and helped develop social capital between them. This was highlighted by the changing willingness of citizens to navigate unfamiliar spaces, to create social ties, and to trust others. The NS helped create a new sense of place for citizens, which ultimately facilitated the creation of social capital in the community. The findings suggest that identities are dynamic and play a role in constructing social capital, as well as who benefits from social capital and who may be excluded. Lessons from this research may inform future social capital interventions.

